# The Clinical Courses and Prognosis of Cirrhotic Patients after First Acute Decompensation: Prospective Cohort Study

**DOI:** 10.3390/diagnostics14010014

**Published:** 2023-12-20

**Authors:** Jung Hee Kim, Sung-Eun Kim, Do Seon Song, Hee Yeon Kim, Eileen L. Yoon, Seong Hee Kang, Young-Kul Jung, Jung Hyun Kwon, Sung Won Lee, Seul Ki Han, Young Chang, Soung Won Jeong, Jeong Ju Yoo, Young-Joo Jin, Gab Jin Cheon, Byung Seok Kim, Yeon Seok Seo, Hyoungsu Kim, Ji Won Park, Tae Hyung Kim, Dong Hyun Sinn, Woo Jin Chung, Hwi Young Kim, Han Ah Lee, Seung Woo Nam, In Hee Kim, Ji Hoon Kim, Hee Bok Chae, Joo Hyun Sohn, Ju Yeon Cho, Jung Gil Park, Hyun Chin Cho, Yoon Jun Kim, Jin Mo Yang, Ki Tae Suk, Moon Young Kim, Sang Gyune Kim, Hyung Joon Yim, Won Kim, Jae-Young Jang, Dong Joon Kim

**Affiliations:** 1 Department of Internal Medicine, Hallym Medical Center, Hallym University College of Medicine, Chuncheon 24252, Republic of Korea; jungheekim@hallym.or.kr (J.H.K.); hskim@kdh.or.kr (H.K.); ktsuk@hallym.ac.kr (K.T.S.); djkim@hallym.ac.kr (D.J.K.); 2 Institute for Liver and Digestive Diseases, Hallym University, Chuncheon 24252, Republic of Korea; 3 Department of Internal Medicine, College of Medicine, The Catholic University of Korea, Seoul 06591, Republic of Korea; dsman@catholic.ac.kr (D.S.S.); hee82@catholic.ac.kr (H.Y.K.); doctorkwon@catholic.ac.kr (J.H.K.); zambrotta@catholic.ac.kr (S.W.L.); jmyangdr@catholic.ac.kr (J.M.Y.); 4 Department of Internal Medicine, Hanyang University College of Medicine, Seoul 04763, Republic of Korea; mseileen80@hanyang.ac.kr (E.L.Y.); sonjh@hanyang.ac.kr (J.H.S.); 5 Department of Internal Medicine, Korea University Medical Center, Seoul 02841, Republic of Korea; shkang14@yonsei.ac.kr (S.H.K.); drseo@korea.ac.kr (Y.S.S.); kjhhepar@naver.com (J.H.K.); gudwns21@korea.ac.kr (H.J.Y.); 6 Department of Internal Medicine, Yonsei University Wonju College of Medicine, Wonju 26426, Republic of Korea; lolhsk@yonsei.ac.kr (S.K.H.); drkimmy@yonsei.ac.kr (M.Y.K.); 7 Department of Internal Medicine, Soonchunhyang University College of Medicine, Seoul 04401, Republic of Korea; chyoung86@gmail.com (Y.C.); jeongsw@schmc.ac.kr (S.W.J.); jyjang@schmc.ac.kr (J.-Y.J.); 8 Department of Internal Medicine, Soonchunhyang University Bucheon Hospital, Bucheon 14584, Republic of Korea; puby17@naver.com (J.J.Y.); mcnulty@schmc.ac.kr (S.G.K.); 9 Department of Internal Medicine, Inha University Hospital, Inha University School of Medicine, Incheon 22212, Republic of Korea; jyj412@hanmail.net; 10 Department of Internal Medicine, Gangneung Asan Hospital, University of Ulsan College of Medicine, Gangneung 25440, Republic of Korea; 1000@gnah.co.kr; 11 Department of Internal Medicine, Daegu Catholic University School of Medicine, Daegu 42472, Republic of Korea; kbs9225@cu.ac.kr; 12 Department of Medicine, Samsung Medical Center, Sungkyunkwan University School of Medicine, Seoul 06531, Republic of Korea; sinndhn@hanmail.net; 13 Department of Internal Medicine, Keimyung University School of Medicine, Daegu 42601, Republic of Korea; chung50@dsmc.or.kr; 14 Department of Internal Medicine, College of Medicine, Ewha Womans University, Seoul 07804, Republic of Koreaamelia86@naver.com (H.A.L.); 15 Department of Internal Medicine, National Medical Center, Seoul 04564, Republic of Korea; kn37503@hotmail.com; 16 Department of Internal Medicine, Chonbuk National University Hospital, Chonbuk National University Medical School, Jeonju 54896, Republic of Korea; ihkimmd@chonbuk.ac.kr; 17 Department of Internal Medicine, Medical Research Institute, Chungbuk National University College of Medicine, Cheongju 28644, Republic of Korea; hbchae@chungbuk.ac.kr; 18 Department of Internal Medicine, College of Medicine, Chosun University, Gwangju 61452, Republic of Korea; jy.cho@chosun.ac.kr; 19 Department of Internal Medicine, Yeungnam University College of Medicine, Daegu 42415, Republic of Korea; gsnrs@naver.com; 20 Department of Internal Medicine, Gyeongsang National University Hospital, Jinju 52727, Republic of Korea; academic77@naver.com; 21 Department of Internal Medicine, Liver Research Institute, Seoul National University College of Medicine, Seoul 03080, Republic of Korea; 22 Department of Internal Medicine, Seoul Metropolitan Government Seoul National University Boramae Medical Center, Seoul 07061, Republic of Korea; wonshiri@yahoo.com

**Keywords:** liver cirrhosis, acute decompensation, prognosis, aetiology

## Abstract

Background: The European Foundation for the Study of Chronic Liver Failure (EF-CLIF) consortium suggested that the clinical courses after acute decompensation (AD) stratify the long-term prognosis: stable decompensated cirrhosis (SDC), unstable decompensated cirrhosis (UDC), pre acute-on-chronic liver failure (pre ACLF), and ACLF. However, previous studies included patients with a history of previous AD and had limitations associated with identifying the clinical factors related to prognosis after the first AD. Method: The prospective Korean Acute-on-Chronic Liver Failure (KACLiF) cohort included cirrhotic patients who were hospitalised with first AD between July 2015 and August 2018. We analysed the factors associated with readmission after the first AD and compared the characteristics and prognosis among each subgroup to evaluate the risk factors for the occurrence of pre ACLF after AD. Result: A total of 746 cirrhotic patients who were hospitalised with first AD were enrolled. The subgroups consisted of SDC (*n* = 565), UDC (*n* = 29), pre ACLF (*n* = 28), and ACLF (*n* = 124). Of note, pre ACLF showed a poorer prognosis than ACLF. The risk factors associated with readmission within 3 months of first AD were non-variceal gastrointestinal (GI) bleeding, hepatic encephalopathy (HE), and high MELD score. Viral aetiology was associated with the occurrence of pre ACLF compared with alcohol aetiology regardless of baseline liver function status. Conclusion: Cirrhotic patients with first AD who present as non-variceal GI bleeding and HE can easily relapse. Interestingly, the occurrence of AD with organ failure within 3 months of first AD (pre ACLF) has worse prognosis compared with the occurrence of organ failure at first AD (ACLF). In particular, cirrhotic patients with viral hepatitis with/without alcohol consumption showed poor prognosis compared to other aetiologies. Therefore, patients with ACLF after AD within 3 months should be treated more carefully and definitive treatment through LT should be considered.

## 1. Introduction

Acute decompensation (AD) is a catastrophic condition for cirrhotic patients, with a poor clinical course that can be accompanied by various events, such as bacterial infection, gastrointestinal (GI) bleeding, alcoholic hepatitis, flare of liver disease, and drug-induced liver injury [1]. Acute-on-chronic liver failure (ACLF), which shows a distinct poor prognosis from AD, is characterised by organ failure and severe systemic inflammation, leading to very high 28-day mortality rates [2,3,4]. The mechanism behind the transition to decompensation and ACLF from compensated cirrhosis is explained by structural changes in the liver, portal hypertension, systemic inflammation, immunodeficiency, and gut dysbiosis [5,6,7].

The CANONIC study, which was conducted by the European Association for the Study of the Liver (EASL)–Chronic Liver Failure (CLIF) Consortium, defined the diagnostic criteria for ACLF using the Chronic Liver Failure Sequential Organ Failure Assessment (CLIF-SOFA) score, and revealed the progression of ACLF with high mortality [3]. Although the physiological mechanisms of ACLF are mostly understood, the development of ACLF and the prediction of its precipitating factors remain areas for further elucidation [6,7]. The recent PREDICT study, which was the second trial for the EASL-CLIF Consortium, described the different clinical courses of AD after hospital admission, combined with different physiology and clinical prognosis, to identify predictors of ACLF [8]. Stable decompensated cirrhosis (SDC) refers to no readmission or ACLF within 3 months after AD, and showing a good prognosis. Unstable decompensated cirrhosis (UDC) refers to readmission within 3 months after AD without ACLF and showing moderate prognosis with severe portal hypertension. Pre ACLF, which includes patients with ACLF within 3 months after AD, shows the worst prognosis with systemic inflammation. Based on this, a CLIF-C ACLF-D score was developed to predict a group of patients with ACLF within 3 months after AD, which included age, ascites, serum white blood cell count, albumin, bilirubin, and creatinine levels. However, they enrolled patients with a previous history of a decompensation event with bacterial infection, ascites, and hepatic encephalopathy (HE), and the proportion of such patients was increased in the readmission groups (UDC and pre ACLF) compared to SDC. These results, including those from patients with a previous history of decompensation, had limitations in evaluating the additional factors that may further affect the prognosis after the first AD. 

In this study, we analysed patients with cirrhosis who experienced their first episode of AD and had no history of previous decompensation events. We validated their prognosis based on clinical courses after 3 months at the time of their first AD, using data from the prospective Korean Acute-on-Chronic Liver Failure (KACLiF) cohort. We also identified the clinical factors that may affect readmission (pre ACLF and UDC) after the first AD. Additionally, we compared the prognosis between the pre ACLF and ACLF groups at admission as a comparator and evaluated the associated factors that could predict the presence of pre ACLF.

## 2. Materials and Methods

### 2.1. Study Subjects

**Figure 1:**
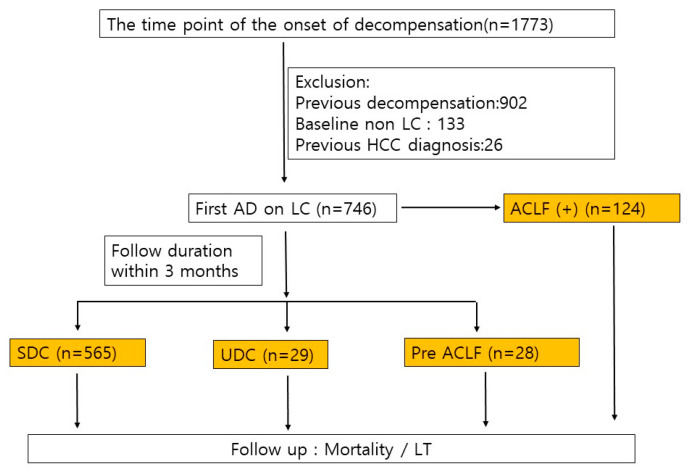
Flow chart for inclusion criteria.

The prospective KACLiF study is a South Korean multicentre, observational study conducted in 23 medical centres, each equipped with a liver unit, liver-patient-specific ward(s), intensive care units, and liver transplantation (LT) programmes. Between July 2015 and August 2018, the 1773 patients who were hospitalised due to acute deterioration of chronic liver disease, encompassing chronic hepatitis and compensated cirrhosis, were included. From this initial pool, we excluded 1061 subjects who met the following criteria: history of previous decompensation (*n* = 902), absence of cirrhosis (*n* = 133), and history of hepatocellular carcinoma (*n* = 26). Ultimately, 746 cirrhotic patients with first AD were identified (Figure 1). To confirm the history of decompensation, we reviewed previous hospital records and patient statements related to liver problems at the time of admission.

Liver cirrhosis (LC) was defined as meeting at least one of the following criteria: (1) radiologic evidence of cirrhotic liver configuration and/or splenomegaly, (2) the presence of varices detected using upper endoscopy or cross-sectional imaging, (3) abnormal biochemical parameters, or (4) historical confirmation. AD was defined as newly developed overt ascites, overt HE, variceal bleeding, non-variceal GI bleeding, any type of bacterial infection, or liver dysfunction deterioration, defined as a serum bilirubin level ≥ 3 mg/dL [9]. This study protocol was approved by the Institutional Review Boards of all 23 participating academic centres. Written informed consent was obtained from patients or their legal surrogates in cases where consent could not be obtained directly from patients prior to enrolment in the study.

### 2.2. Data Collection and Definitions of Clinical Parameters

Data on patient demographics, the aetiology of liver disease, clinical and laboratory variables, type of AD, precipitating events, and the development of ACLF were collected. The viral aetiology related to chronic liver disease (CLD) and autoimmune-related CLD was defined based on definitive viral serology and biochemical parameters and/or liver pathology as diagnostic criteria [10,11,12]. The aetiology for alcohol-related CLD was defined in patients who had consumed more than moderate amounts of alcohol (14 units/week for men and 7 units/week for women) with alcohol use disorder at the time of the diagnosis of CLD [13,14]. Precipitating events included any kind of bacterial infection, variceal bleeding, non-variceal GI bleeding, active alcoholism, reactivation of viral hepatitis, toxic liver injury, and others. Systemic inflammatory response syndrome (SIRS) was defined according to the criteria of the American College of Chest Physicians/Society of Critical Care Medicine [15]. The Child–Turcotte–Pugh score (CPS) and Model for End-Stage Liver Disease (MELD) score were calculated based on the clinical variables within 24 h of admission. Patients who developed AD and organ failure were classified as having ACLF according to the CLIF-C definition [16]. The clinical course of patients without ACLF after the first AD was divided into three stages during a 3-month follow-up period: SDC, including patients without ACLF or readmission within the follow-up period; UDC, including patients who experienced at least one readmission without ACLF within 3 months; and pre ACLF, including patients who developed ACLF within 3 months of their first AD. 

### 2.3. Primary Outcomes and Follow-Up

The primary endpoints of this study were 90-day and 1-year LT-free mortality during the follow-up period. Adverse outcomes were defined as death or liver transplantation. All participants were followed up until one of the following conditions: last hospital visit, liver transplantation, death, or the end date of the study (31 May 2019), whichever occurred first.

### 2.4. Statistical Analyses

Continuous variables were presented as means ± standard deviations or medians with ranges, and were compared using the Student’s *t*-test or Mann–Whitney U test. Discrete variables were reported as the number of events and percentages for each category and compared using the appropriate statistical tests, such as the χ^2^ test or Fisher’s exact test. The Kaplan–Meier method was used to estimate survival curves, and differences between groups were compared using the log-rank test. Logistic regression analysis was performed to determine the factors associated with readmission within 3 months of first AD and the presence of pre ACLF among the patients with first AD. Furthermore, multiple logistic regression analysis was conducted using variables that were associated with the outcome in univariate analysis with a *p*-value < 0.1. A *p*-value < 0.05 was considered statistically significant. Statistical analyses were performed using SPSS for Windows, version 27.0 (SPSS Inc., Chicago, IL, USA).

## 3. Results

### 3.1. Study Population and Baseline Characteristics According to Clinical Courses after the First Acute Decompensation

Among the 1773 patients with AD, 746 cirrhotic patients were enrolled for the first AD, and among them, 124 cirrhotic patients had ACLF (Figure 1). Without ACLF at admission, 622 patients were observed for 3 months after the first AD. Of these, 565 patients (90.8%) were observed in the SDC group, while 57 patients (9.2%) were observed in the readmission group within 3 months after the first AD (29 in the UDC group and 28 in the pre ACLF group, respectively). 

The baseline characteristics of the 622 patients with the first AD are shown in Table 1. The median follow-up was 10.0 months (3.0–16.0 months), the mean age was 54.9 years, and males accounted for 72.2%. The most common aetiology was alcohol (70.0%), followed by hepatitis B virus (HBV) or hepatitis C virus (HCV) (11.3%) and HBV or HCV with alcohol (10.6%). The most common clinical presentation of AD was jaundice (36.8%), followed by ascites (32.5%) and varix bleeding (29.7%). The group of patients with readmission within 3 months (UDC and pre ACLF) showed an increased incidence of AD events such as non-variceal bleeding and HE compared with the SDC group. They also had worse profiles in terms of CPS, MELD, and CLIF-C AD score compared with the SDC group at the first AD. However, there was no significant difference between alcohol consumption/amount, aetiology, and the presence of SIRS between the SDC group and the readmission group (UDC and pre ACLF).

### 3.2. Short/Long-Term Mortality According to Clinical Course and the Factors Associated with Readmission in Patients after the First Acute Decompensation

**Figure 2:**
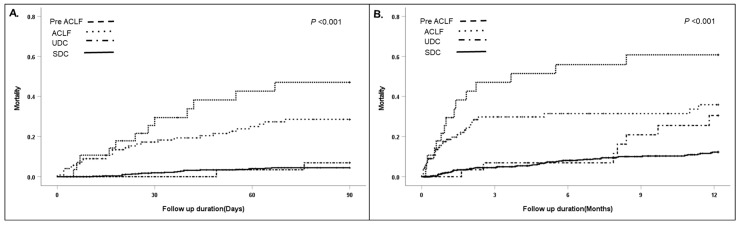
(**A**) Comparison of 90-day mortality according to clinical course after first AD. (**B**) Comparison of 1-year mortality according to the clinical course after first AD.

We evaluated the short- and long-term mortality of patients after the first AD without ACLF according to their clinical course (Table 1). Patients in the UDC and pre ACLF groups with readmission had significantly worse 90-day and 1-year mortality rates compared to those in the SDC group (90-day: SDC = 3.9%, UDC = 6.9%, and pre ACLF = 42.9%, *p* < 0.001; 1-year: SDC = 11%, UDC = 27.6%, and pre ACLF = 54.6%) (Figure 2). The majority of patients who underwent LT during the follow-up period were in the SDC group (Table 1). The pre ACLF group exhibited the highest mortality, followed by the UDC and SDC groups in both the low and high MELD tier (Table 2).

We analysed the risk factors associated with readmission (pre ACLF and UDC) in cirrhotic patients after the first AD (Table 3). Non-variceal GI bleeding, HE, serum albumin level, serum Na level, and MELD score were associated with readmission in the unadjusted analysis. In the adjusted analysis, non-variceal GI bleeding was found to be associated with readmission after the first AD along with initial liver function status such as MELD score CLIF AD score.

### 3.3. Clinical Difference between Initial ACLF at First AD and Newly Developed ACLF within 3 Months of First AD (Pre ACLF)

**Figure 3:**
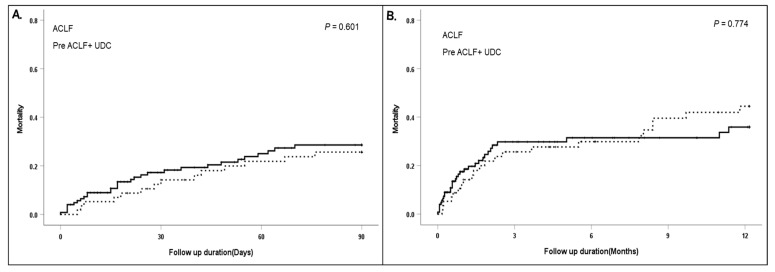
(**A**) Comparison of 90-day mortality between the readmission (pre ACLF + UDC) and ACLF groups. (**B**) Comparison of 1-year mortality between the readmission (pre ACLF + UDC) and ACLF groups. ACLF: dotted line; Pre ACLF + UDC: solid line.

The cohort included 124 patients with ACLF at first AD. Comparing the prognosis between readmission after first AD (UDC and pre ACLF) and the ACLF group, we found that the readmission groups and the ACLF group showed comparable outcomes (Figure 3). However, the ACLF group had a worse 90-day/1-year mortality rate than the SDC/UDC groups, but better survival than the pre ACLF group (Figure 2). To evaluate the factors associated with the occurrence of pre ACLF, we compared the clinical characteristics and adverse outcomes between the ACLF and pre ACLF groups (Table 4).

Both the ACLF and pre ACLF groups had alcohol-related CLD as the most common aetiology. However, chronic viral hepatitis and viral with alcohol-related CLD were more prevalent in the pre ACLF group than in the ACLF group. Viral activation among the precipitating events was significantly increased in the pre ACLF group, associated with viral hepatitis as a major aetiology. Non-variceal GI bleeding was a common event at first AD in the pre ACLF group, and its incidence was significantly higher than that in the ACLF group. The alcohol consumption and amount values were higher in the ACLF group than in the pre ACLF group, and the MELD score at admission was also higher in the ACLF group than in the other groups. During the follow-up period, six patients (4.8%) received LT in the ACLF group within 3 months, but none in the pre ACLF group, despite its high mortality.

To evaluate the factors associated with the presence of pre ACLF, we compared the baseline characteristics among patients with first AD (Table 5). In an adjusted analysis of two models, a high MELD score, CLIF AD score, and non-variceal GI bleeding at first AD were associated with the presence of pre ACLF among the patients with first AD. Moreover, viral aetiology and viral and alcohol aetiology were more positively associated with the presence of pre ACLF than alcohol aetiology in both models considering MELD or CLIF AD scores. 

## 4. Discussion

Recurrent liver injury, which is attributable to various predisposing and precipitating factors, can promote the progression of compensation status to decompensation in cirrhotic patients. In this regard, efforts to evaluate prognosis and identify the factors associated with recurrent liver injury resulting in hospitalisation or ACLF are necessary to optimise management strategies for cirrhotic patients. In this study, we compared and validated the different clinical courses after the first AD and identified the factors associated with readmission (UDC and pre ACLF) after the first AD. We also compared the prognosis between pre ACLF and ACLF and identified the factors associated with the presence of ACLF within 3 months of the first AD.

The prognosis after the first AD was stratified by different clinical courses divided into with/without readmission (SDC, UDC, and pre ACLF) and ACLF. In our cohort, which included a high proportion of alcohol-related CLD, the baseline MELD was higher than in the PREDICT study, which included patients with a history of previous decompensation (MELD = 21.5 versus MELD = 19.5 in the PREDICT cohort) [8]. This was because our cohort had a higher proportion (over 70%) of alcohol-related CLD cases than the PREDICT study. Alcohol-related liver damage is complex and multifactorial, involving both oxidative stress and cytotoxicity in the liver, and weakens the immune response to the hepatitis virus. Additionally, alcohol and viral hepatitis have a negative synergistic effect on patients, accelerating the progression of liver damage in addition to the toxicity of the alcohol itself [17]. The AD of alcohol-related liver disease often shows SIRS, even in the absence of infection. SIRS, with or without infection, is a major determinant of multi-organ failure and mortality in alcoholic hepatitis [18].

The stratification of prognosis according to different clinical courses after AD was consistent between the two cohorts, regardless of its aetiology, even though our cohort included cirrhotic patients without a history of decompensation. Poor liver function at admission, including a high MELD score, was associated with a readmission course (UDC and pre ACLF), which induced a poor prognosis compared to SDC in our study. MELD scores have been shown to be associated with 90-day mortality from various liver diseases, and the allocation algorithm using the MELD score has been shown to reduce LT waiting list mortality and improve patient survival [19,20]. It is now being extended to evaluate patients with complications of cirrhosis or mortality from major interventions, such as non-transplant surgery in patients with cirrhosis [6,7,8]. However, despite its advantages, the MELD score incorrectly predicts mortality in about 15–20% of patients due to the score not including major cirrhotic complications such as bleeding, bacterial infection, HE, and albumin levels, which induce a poor prognosis for patients [9]. In a subgroup analysis of our study, the clinical courses of SDC, UDC, and pre ACLF stratified the prognosis in both groups with low and high MELD scores. This implies that not only baseline liver function, but also other readmission-related factors, such as predisposing or precipitating factors, may crucially affect the prognosis after the first AD. 

Previously, robust indicators for the prognosis of decompensation included clinical parameters combined with quantitative measures such as CPS, which includes components such as albumin, bilirubin, ascites, encephalopathy, and prothrombin time, and the measurement of hepatic vein pressure gradient with MELD score [21,22]. Knowledge about the pathophysiologic mechanisms of AD/ACLF caused by hepatic damage, portal hypertension, systemic inflammation, immunodeficiency, and gut symbiosis has also helped predict prognosis and identify related factors such as the CLIF-C ACLF-D score, which is a tool to predict the development of ACLF with higher accuracy than MELD and CPS and is composed of age, ascites, WBC count, albumin, bilirubin, and creatinine levels [5,8].

Th other associated clinical factors for readmission courses (UDC and pre ACLF) compared to SDC in our study were non-variceal GI bleeding and HE. In a meta-analysis for non-variceal GI bleeding in cirrhotic patients, the most common causes of upper GI bleeding except variceal bleeding were portal hypertensive gastropathy (PHG) (20–98%) and peptic ulcer disease (40–50%) [23,24,25]. In a previous case–control study of 294 cirrhotic patients, the origin (variceal vs. non-variceal) of GI bleeding in cirrhotic patients did not affect the development of other complications and mortality, except for acute renal injury in variceal bleeding [26]. However, non-variceal GI bleeding with PHG and peptic ulcer disease in cirrhosis patients was difficult to control compared to varix bleeding due to limitations in the use of direct endoscopy therapy in diffuse patterned PHG and poor wound healing due to decreased gastrointestinal mucosal flow [27]. This can cause recurrent acute or chronic GI bleeding, induce additional hepatic insult than other tolerable and adjustable events of AD, and lead to readmission after the first AD.

HE is a brain dysfunction caused by liver insufficiency and/or portosystemic shunts and is characterised by various grades of severity. The pathophysiology of HE is multifactorial and complex, meaning that multiple aetiological factors exist and are difficult to control [28]. The occurrence of HE during AD is related to poor prognosis, independently of the severity of cirrhosis in patients with AD/ACLF, compared to ascites and variceal bleeding [8]. Additionally, HE has been associated with a significant impact on patients’ health-related quality of life, and changes in mental status are easily noticeable and can lead to readmission.

We also evaluated the clinical difference between ACLF at first AD and ACLF after first AD within 3 months (pre ACLF). Interestingly, the initial MELD score at first AD was higher in ACLF than pre ACLF, but the prognosis for 90-day and 1-year mortality was poor in the pre ACLF group. It is considered that the patients with ACLF at first AD were aggressively and more completely controlled for precipitating events (such as alcohol consumption) than those with pre ACLF, which progressed hepatic insult through intractable triggering events. These findings were also observed in a real-life retrospective observational study of 222 cirrhotic patients comparing clinical courses after AD [29]. In our study, a high MELD score or CLIF AD score was associated with the presence of pre ACLF, and the viral aetiology also showed a positive association with the presence of pre ACLF. HCV-infected patients treated with direct-acting antivirals reach a sustained virological response rate in 2–3 months, and HBV-infected patients treated with nucleoside/nucleotide agents achieve at least viral suppression within almost one year [10,30]. Previously, a prospective study evaluating the prognosis of HBV-ACLF, including patients with a history of decompensation, showed that the short-term mortality rate of cirrhotic HBV-ACLF was significantly higher than the CANONIC group, which was predominantly alcoholic cirrhosis [31]. Even if cirrhotic patients with viral hepatitis were hospitalised for AD without initial ACLF, they should be promptly started on antiviral agents, carefully observed, and consider aggressive management, including LT, to prevent the occurrence of ACLF within 3 months (pre ACLF) after the first AD.

This study has some limitations. The study was conducted using hospitalised patients with AD and did not include patients who only visited outpatient clinics with mild AD symptoms. The transition from compensation to decompensation also includes patients who have mild ascites, jaundice, and mild HE, who do not require hospitalisation but receive medical treatment. In this regard, our study sample may not be entirely representative of the general population due to potential selection bias. Since the number of patients in the pre ACLF group was relatively small compared to other groups, a large prospective observational study according to aetiology will be needed to further validate the evidence of this study.

## 5. Conclusions

In conclusion, patients readmitted after the first AD (UDC and pre ACLF) had a worse prognosis compared to patients without readmission (SDC). The occurrence of ACLF within 3 months of first AD (pre ACLF) had a worse prognosis compared with the occurrence of ACLF at first AD. Cirrhotic patients with first AD should be cautious of readmission due to HE and non-variceal bleeding, which are difficult to control and can easily recur. In particular, patients with first AD that developed from viral hepatitis should be carefully monitored for the occurrence of the ACLF within 3 months of the first AD (pre ACLF), and definitive treatment through LT may also need to be considered. A large-scale global study is necessary to investigate the prognostic difference according to the aetiology of liver disease and to validate the clinical factors in patients who experience further decompensation after their first AD.

## Figures and Tables

**Table 1:** Baseline characteristics and outcomes at the date of first decompensation according to different clinical course.

	Total (*n* = 622)	SDC (*n* = 565)	Readmission within 3 Months at First AD		*p*-Value
			UDC (*n* = 29)	Pre ACLF (*n* = 28)	
Baseline characteristics at first AD					
Age (years)	54.9 11.6	54.7 11.3	58.9 14.0	54.7 13.0	0.258
Male (%)	449 (72.2)	412 (72.9)	19 (65.5)	18 (64.3)	0.199
Aetiology					0.197
Virus	92 (12.3)	77 (13.6)	1 (3.4)	9 (32.1)	
Alcohol	513 (68.8)	381 (67.4))	20 (69.0)	13 (46.4)	
Virus and Alcohol	75 (10.1)	55 (9.7)	2 (6.9)	5 (17.9)	
AIH/PBC	25 (3.4)	22 (3.9)	2 (6.9)	0	
Cryptogenic	41 (6.5)	30 (5.3)	4 (13.8)	1 (3.6)	
AD					
Ascites	202 (32.5)	185 (32.7)	9 (31.0)	8 (28.6)	0.654
Bacterial infection	44 (7.1)	37 (6.5)	5 (17.2)	2 (7.1)	0.108
Varix bleeding	185 (29.7)	172 (30.4)	8 (27.6)	5 (17.9)	0.230
Nonvariceal bleeding	46 (7.4)	37 (6.5)	3 (10.3)	6 (21.4)	0.011
HE	49 (7.9)	40 (7.1)	5 (17.2)	4 (14.3)	0.020
Jaundice	229 (36.8)	210 (37.2)	8 (27.6)	11 (39.3)	0.568
CKD	6 (1.0)	4 (0.7)	1 (3.4)	1 (3.6)	1.000
DM	132 (21.2)	120 (21.2)	6 (20.7)	6 (21.4)	1.000
HTN	135 (21.7)	120 (21.2)	7 (24.1)	8 (28.6)	1.000
PE					
Alcoholism	322 (51.8)	296 (52.4)	11 (37.9)	15 (53.6)	0.330
Bacterial infection	30 (4.8)	26 (4.6)	3 (10.3)	1 (3.6)	0.418
Varix bleeding	127 (20.4)	116 (20.5)	8 (27.6)	3 (10.7)	0.826
Non-variceal bleeding	32 (5.1)	25 (4.4)	2 (6.9)	5 (17.9)	0.011
Toxic	12 (1.9)	11 (1.9)	0	1 (3.6)	0.920
Virus activation	26 (4.2)	21 (3.7)	0	5 (17.9)	0.069
Others	28 (4.5)	25 (4.4)	0	0	0.771
Alcohol intake #	428 (68.8)	394 (69.7)	18 (62.1)	16 (57.1)	0.108
Alcohol amount (g/day)	50.0 (0–100)	50.0 (0–100)	50 (0–100)	19.2 (0–75.0)	0.261
SIRS, *n* (%)	127 (25.2)	142 (25.1)	4 (13.8)	11 (39.3)	0.845
Laboratory data					
WBCx103/L	7.15 (5.00–10.26)	7.20 (5.00–10.32)	6.72 (5.10–9.14)	6.85 (5.50–11.23)	0.600
Haemoglobin	10.9 (8.7–12.5)	10.9 (8.7–12.5)	10.3 (7.3–12.4)	10.7 (8.4–12.7)	0.395
Platelet, mg/L	103 (70–151)	105 (70–151)	111 (76–155)	76 (54–137)	0.246
Bilirubin, mg/dL	3.3 (1.5–7.5)	3.3 (1.5–7.3)	3.2 (1.2–5.7)	10.0 (2.3–15.9)	0.071
Albumin, g/dL	2.9 (2.6–3.3)	2.9 (2.6–3.3)	2.8 (2.6–3.3)	2.8 (2.3–3.1)	0.159
INR	1.43 (1.25–1.71)	1.42 (1.24–1.70)	1.39 (1.25–1.59)	1.76 (1.54–2.23)	0.006
Creatinine, mg/dL	0.8 (0.6–1.0)	0.8 (0.6–1.0)	0.9 (0.6–1.2)	0.7 (0.6–1.1)	0.852
Sodium, mEq/L	137 (133–140)	137 (133–140)	136 (133–139)	133 (130–137)	0.021
Child–Pugh score	9.0 (7.0–10.0)	9.0 (7.0–10.0)	9.0 (7.5–11.0)	10.0 (9.0–11.0)	0.010
MELD score	15.6 (11.9–20.4)	15.5 (11.7–20.2)	15.0 (10.9–18.5)	21.5 (15.0–25.5)	0.017
MELD-Na score	17.9 (13.6–23.7)	17.7 (13.3–23.3)	17.3 (14.0–23.0)	25.1 (18.5–28.5)	0.007
MELD-3 score	14.2 (8.1–20.3)	14.0 (7.8–19.7)	14.8 (7.8–19.5)	21.7 (16.4–25.0)	0.003
CLIF-C AD	57.7 (52.3–63.6)	57.2 (52.1–63.3)	60.4 (23.0–64.9)	62.8 (59.2–66.9)	0.007
Clinical course after first AD					
Hospitalisation < 3 month					
1	53 (8.5)	0	28 (96.6)	25 (89.3)	
2	4 (0.6)	0	1 (3.4)	3 (10.7)	
Adverse outcomes					
90-day mortality	36 (5.8)	22 (3.9)	2 (6.9)	12 (42.9)	<0.001
LT	9 (1.4)	8 (1.4)	1 (3.4)	0	
1-year mortality	73 (11.7)	51 (9.0)	7 (24.1)	15 (53.6)	<0.001
LT	12 (1.9)	11 (1.9)	1 (3.4)	0	
Overall mortality	90 (14.5)	65 (11.5)	9 (31.0)	16 (57.1)	<0.001
LT	12 (1.9)	11 (1.9)	1 (3.4)	0	

AD, acute decompensation; SDC, stable decompensated cirrhosis; UDC, unstable decompensated cirrhosis, Pre ACLF, pre acute-on-chronic liver failure; AIH, autoimmune hepatitis; PBC, primary biliary cholangitis; HE, hepatic encephalopathy; CKD, chronic kidney disease; HTN, hypertension; DM, diabetic mellitus; SIRS, systemic inflammatory response syndrome; WBC, white blood cell; INR, international normalised ratio; MELD, Model for End-Stage Liver Disease; CLIF-C AD, CLIF Consortium Acute Decompensation score; LT, liver transplantation; # Current alcohol intake within 3 months.

**Table 2:** Subgroup analysis for MELD score.

	90-Day Mortality (%)			*p*	1-Year Mortality (%)			*p*
	SDC	UDC	Pre ACLF		SDC	UDC	Pre ACLF	
MELD, initial								
<15 (*n* = 282)	2/262 (0.8)	1/14 (7.1)	1/6 (16.7)	0.003	8/262 (3.1)	2/14 (14.3)	3/6 (50.0)	<0.001
≥15 (*n* = 339)	23/302 (9.3)	2/15 (13.3)	11/22 (50.0)	<0.001	54/302 (17.9)	6/15 (40)	12/22 (54.5)	<0.001

SDC, stable decompensated cirrhosis; UDC, unstable decompensated cirrhosis, Pre ACLF, pre acute-on-chronic liver failure; MELD, Model for End-Stage Liver Disease.

**Table 3:** Factors associated with readmission within 3 months of first AD.

	Unadjusted OR (95% CI)	*p*-Value	Adjusted OR (95% CI) *p*-Value			
			Model 1	*p*-Value	Model 2	*p*-Value
Age	1.016 (0.992–1.039)	0.192				
Sex	0.687 (0.387–1.221)	0.200				
Aetiology of LC	1.095 (0.829–1.448)	0.522				
Ascites	0.873 (0.482–1.581)	0.654				
Bacterial infection	1.998 (0.847–4.713)	0.114				
Varix bleeding	0.675 (0.354–1.286)	0.232				
Non-variceal bleeding	2.676 (1.219–5.873)	0.014	3.089 (1.376–6.935)	0.006	2.747 (1.236–6.103)	0.013
HE	2.461 (1.127–5.375)	0.024	2.858 (1.273–6.388)	0.011	2.532 (1.144–5.602)	0.022
Jaundice	0.845 (0.475–1.504)	0.568				
Haemoglobin	0.962 (0.874–1.058)	0.422				
Platelet	0.999 (0.995–1.003)	0.566				
Total bilirubin	1.039 (1.004–1.075)	0.027				
Albumin	0.656 (0.411–1.048)	0.078				
INR	1.971 (1.068–3.637)	0.030				
Na	0.959 (0.918–1.002)	0.060				
Alcohol intake	0.634 (0.363–1.109)	0.110				
Alcohol amount	0.998 (0.994–1.002)	0.277				
SIRS	1.064 (0.573–1.977)	0.845				
CLIF AD score	1.058 (1.015–1.103)	0.008			1.034 (1.002–1.067)	0.035
MELD-Na	1.064 (1.019–1.112)	0.005	1.075 (1.027–1.125)	0.002		

Model 1 included the non-variceal bleeding, HE, and MELD-Na; Model 2 included the non-variceal bleeding, HE, and CLIF AD score. OR, odds ratio; CI, confidence interval; LC, liver cirrhosis; HE, hepatic encephalopathy; INR, international normalised ratio; SIRS, systemic inflammatory response syndrome; CLIF-C AD, The CLIF Consortium Acute Decompensation score; MELD, Model for End-Stage Liver Disease.

**Table 4:** Comparison of the baseline characteristics and outcomes at the date of first decompensation between pre ACLF and ACLF groups.

	Pre ACLF (*n* = 28)	ACLF (*n* = 124)	*p*-Value
Baseline characteristics at first AD			
Age (years)	54.7 13.0	53.6 10.2	0.676
Male (%)	18 (64.3)	103 (83.1)	0.026
Aetiology			0.042
Virus	4 (14.3)	5 (4.0)	
Alcohol	20 (71.4)	99 (79.8)	
Virus and Alcohol	3 (10.7)	13 (1.5)	
AIH/PBC	0	1 (0.8)	
Cryptogenic	1 (3.6)	6 (4.8)	
AD			
Ascites	8 (28.6)	32 (25.8)	0.765
Bacterial infection	2 (7.1)	14 (11.3)	0.520
Varix bleeding	5 (17.9)	28 (22.6)	0.585
Non-variceal bleeding	6 (21.4)	8 (6.5)	0.014
HE	4 (14.3)	38 (30.6)	0.081
Jaundice	11 (39.3)	61 (49.2)	0.345
CKD	1 (3.6)	11 (8.9)	1.000
DM	6 (21.4)	28 (22.6)	1.000
HTN	8 (28.6)	27 (21.8)	1.000
PE			
Alcoholism	15 (53.6)	84 (67.7)	0.157
Bacterial infection	1 (3.6)	13 (10.5)	0.255
Varix bleeding	3 (10.7)	19 (15.3)	0.533
Non-variceal bleeding	5 (17.9)	7 (5.6)	0.031
Toxic	1 (3.6)	1 (0.8)	0.248
Virus activation	5 (17.9)	1 (0.8)	<0.001
Others	0	2 (1.6)	0.500
Alcohol intake #	16 (57.1)	101 (81.5)	0.006
Alcohol amount (g/day)	19.2 (0–75.0)	75 (35.0–142.5)	0.006
SIRS, *n* (%)	11 (39.3)	47 (37.9)	0.892
Laboratory data			
WBCx103/L	6.85 (5.50–11.23)	9.56 (6.84–13.12)	0.029
Haemoglobin	10.7 (8.4–12.7)	10.3 (8.1–12.2)	0.275
Platelet, mg/L	76 (54–137)	92 (59–142)	0.358
Bilirubin, mg/dL	10.0 (2.3–15.9)	7.8 (2.9–19.6)	0.888
Albumin, g/dL	2.8 (2.3–3.1)	2.7 (2.2–3.0)	0.499
INR	1.76 (1.54–2.23)	1.84 (1.36–2.76)	0.408
Creatinine, mg/dL	0.7 (0.6–1.1)	2.1 (1.2–3.0)	<0.001
Sodium, mEq/L	133 (130–137)	133 (130–137)	0.973
Child–Pugh score	10.0 (9.0–11.0)	11.0 (9.0–12.0)	0.083
MELD score	21.5 (15.0–25.5)	28.0 (22.1–34.0)	<0.001
MELD-Na score	25.1 (18.5–28.5)	30.4 (25.0–35.7)	<0.001
ACLF grade			0.461
1	9 (32.1)	56 (45.2)	
2	15 (53.6)	45 (36.3)	
3	4 (14.3)	23 (18.5)	
Clinical course after first AD			
Hospitalisation < 3 month			<0.001
1	25 (89.3)	3 (2.4)	
2	3 (10.7)	1 (0.8)	
Adverse events			
90-day mortality	12 (42.9)	30 (24.2)	0.047
LT	0	6 (4.8)	
1-year mortality	15 (53.6)	33 (26.6)	0.006
LT	0	6 (4.8)	

AD, acute decompensation; SDC, stable decompensated cirrhosis; UDC, unstable decompensated cirrhosis, Pre ACLF, pre acute-on-chronic liver failure; AIH, autoimmune hepatitis; PBC, primary biliary cholangitis; HE, hepatic encephalopathy; CKD, chronic kidney disease; HTN, hypertension; DM, diabetic mellitus; SIRS, systemic inflammatory response syndrome; WBC, white blood cell; INR, international normalised ratio; MELD, Model for End-Stage Liver Disease; CLIF-C AD, CLIF Consortium Acute Decompensation score; LT, liver transplantation; # Current alcohol intake within 3 months.

**Table 5:** Factors associated with the presence of pre ACLF among the patients with first AD.

	Unadjusted OR (95% CI)	*p*-Value	Adjusted OR (95% CI)			
			Model 1	*p*-Value	Model 2	*p*-Value
Age	0.998 (0.966–1.032)	0.925				
Sex	0.681 (0.308–1.506	0.342				
Aetiology of LC		0.012		0.001		0.005
Alcohol	1		1		1	
Virus and Alcohol	2.706 (0.930–7.878)	0.068	3.235 (1.056–9.970)	0.040	2.964 (0.990–8.876)	0.052
Virus	3.559 (1.471–8.614)	0.005	5.535 (2.125–14.419)	<0.001	4.294 (1.719–10.724)	0.002
Ascites	0.825 (0.327–1.906)	0.652				
Bacterial infection	1.011 (0.232–4.406)	1.011				
Varix bleeding	0.500 (0.187–1.336)	0.167				
Non-variceal bleeding	3.777 (1.449–9.846)	0.007	5.536 (1.763–17.380)	0.003	3.420 (1.165–10.038)	0.025
HE	2.033 (0.676–6.116)	0.207				
Jaundice	1.116 (0.513–2.426)	0.782				
CKD	4.363 (0.493–38.651)	0.186				
DM	1.013 (0.402–2.552)	0.978				
HTN	1.471 (0.633–3.417)	0.370				
Haemoglobin	1.032 (0.900–1.183)	0.652				
Platelet	0.996 (0.989–1.002)	0.206				
Total bilirubin	1.087 (1.045–1.130)	<0.001				
Albumin	0.494 (0.250–0.975)	0.042				
INR	4.459 (2.144–9.274)	<0.001				
Cr	0.867 (0.247–3.047)	0.824				
Na	0.946 (0.893–1.002)	0.058				
Alcohol intake	0.583 (0.270–1.256)	0.168				
Alcohol amount	0.996 (0.990–1.002)	0.230				
SIRS	1.985 (0.909–4.336)	0.085				
MELD-Na	1.154 (0.179–1.234)	<0.001	1.198 (1.108–1.295)	<0.001		
CLIP AD score	1.058 (1.015–1.103)	0.008			1.072 (1.024–1.122)	0.003

Model 1 included the aetiology of LC, non-variceal bleeding, and MELD-Na; Model 2 included the aetiology of LC, non-variceal bleeding, and CLIF AD score. OR, odds ratio; CI, confidence interval; LC, liver cirrhosis; HE, hepatic encephalopathy; INR, international normalised ratio; SIRS, systemic inflammatory response syndrome; CLIF-C AD, CLIF Consortium Acute Decompensation score; MELD, Model for End-Stage Liver Disease.

## Data Availability

The data are unavailable due to privacy or ethical restrictions.
